# Crystal structure of di-μ-acetato-κ^4^*O*:*O′*-bis{(acetato-κ^2^*O*,*O*′)tetra­aqua­[1-(pyridin-2-yl­methylidene-κ*N*)-2-(pyridin-2-yl-κ*N*)hydrazine-κ*N*^1^]lanthanum(III)} dinitrate hemihydrate

**DOI:** 10.1107/S2056989024012349

**Published:** 2025-01-01

**Authors:** Mbossé Ndiaye-Gueye, Bocar Traoré, Ibrahima Elhadji Thiam, Ousmane Diouf, Emmanuel Wenger, Abdou Salam Sall, Claude Lecomte, Mohamed Gaye

**Affiliations:** a Département de Chimie Faculté des Sciences et Techniques Université Cheik Anta Diop Dakar Senegal; b CRM2 Université de Lorraine and CNRS Vandoeuvre Les Nancy France; University of Kentucky, USA

**Keywords:** lanthanum, crystal structure, 2-hydrazino­pyridine,hydrazone

## Abstract

In the binuclear title complex, [La_2_(C_2_H_3_O_2_)_4_(C_11_H_10_N_4_)(H_2_O)_4_](NO_3_)_2_·0.5H_2_O, the two lanthanum ions are nine coordinate in a distorted trigonal–prismatic geometry. Each La^III^ ion is bonded to three N atoms of the Schiff base, 1-(pyridin-2-yl)-2-(pyridin-2-yl­methyl­ene)hydrazine and is coordinated by one acetate group, which acts in *η*^2^-bidentate mode and two acetate groups that act in *μ*_2_-mode between the two La^III^ ions. Two *η*^1^-water mol­ecules complete the coordination sphere. All bond lengths in the coordination environment of the La^III^ ion are slightly larger than those observed in the isostructural Nd^III^ and Sm^III^ complexes. The La^III^⋯La^III^ distance is 4.6696 (6) Å. In the crystal, extensive O—H⋯O hydrogen-bonding inter­actions involving the coordinated water mol­ecules and the non-coordinating nitrate anions, as well as the oxygen atoms of the acetate groups, generate an overall three-dimensional supra­molecular network.

## Abstract 2

In the title Schiff base binuclear lanthanum(III) complex, the two metal ions have the same environment and the La^III^ ion is coordinated by three soft nitro­gen atoms from the Schiff base ligand, four hard oxygen atoms from carboxyl­ate co-ligands and two water oxygen atoms. Each La^III^ ion is nine coordinate and its environment is best described as a tricapped trigonal–prismatic geometry.

## 1. Chemical context

Lanthanide–Schiff base complexes are widely used in applied and fundamental sciences. Chemists continue to pay much attention in the preparation of functional Schiff bases and their lanthanide complexes, which can be used in many fields such as catalysis (Bell *et al.*, 2022 ▸), radiopharmaceuticals (Hu & Wilson, 2022 ▸), fluoro­immuno assay reagents (Wu *et al.*, 2024 ▸; Dong *et al.*, 2023 ▸), diagnostic tools in biology (Liu *et al.*, 2020 ▸; Zapolotsky *et al.*, 2022 ▸), and in laser development (Lapaev *et al.*, 2019 ▸). The use of acyclic Schiff bases allows the introduction of two identical or different metal ions (Geng *et al.*, 2022 ▸; Bryleva *et al.*, 2023 ▸). The presence of multiple coordination sites and the versatile coordination modes provide several possible structures with lanthanide ions (Le Fur *et al.*, 2018 ▸; Kariaka *et al.*, 2019 ▸). Organic ligands that are used as precursors for the structural design of complexes can have hard and/or soft sites such as oxygen, nitro­gen or sulfur atoms. Through proper design, the mol­ecular structure of the ligand can be controlled to have suitable sites to coordinate metal ions to generate specific architectures. The introduction of co-ligands offers multiple possibilities to develop original structures. Carboxyl­ate groups are versatile co-ligands, which can adopt various coordination modes, to generate different structures with the same ligand (Grebenyuk *et al.*, 2021 ▸; Wang *et al.*, 2012 ▸). However, lanthanides can have high and variable coordination numbers, depending on the synthesis conditions of the complexes. Indeed, the synthesis of these compounds is considerably influenced by the reaction procedures and conditions such as the nature of the solvent, pH, temperature and/or reaction time (Sinchow *et al.*, 2019 ▸). This provides a versatility in coordination geometries that makes it difficult to predict the structures and properties of lanthanide com­pounds. In this context, for the synthesis of lanthanide(III) complexes, the Schiff base 1-(pyridin-2-yl­methyl­idene)-2-(pyridin-2-yl)hydrazine (H*L*), which provides three soft donor N atoms from two pyridine rings and an azomethine unit, was used in the presence of acetate anions as co-ligands, which provide hard donor O atoms. Several complexes from the ligand H*L* have been reported by our group (Gueye, Dieng *et al.*, 2017 ▸; Ndiaye-Gueye, Dieng, Thiam, Sow *et al.*, 2017 ▸; Sarr *et al.*, 2018 ▸). In all of these complexes, the acetate group is either bidentate chelating *η*^2^-OOCH_3_, bridging *μ*_2_-OOCH_3_ or bidentate bridging *η*^2^:*μ*_2_-OOCH_3_. This report presents the synthesis, characterization, and X-ray structure of a lanthanum (III) complex derived from 1-(pyrydin-2­yl)-2-(pyridine-2-yl­methyl­ene)hydrazine (H*L*) and an acetate group as co-ligand.
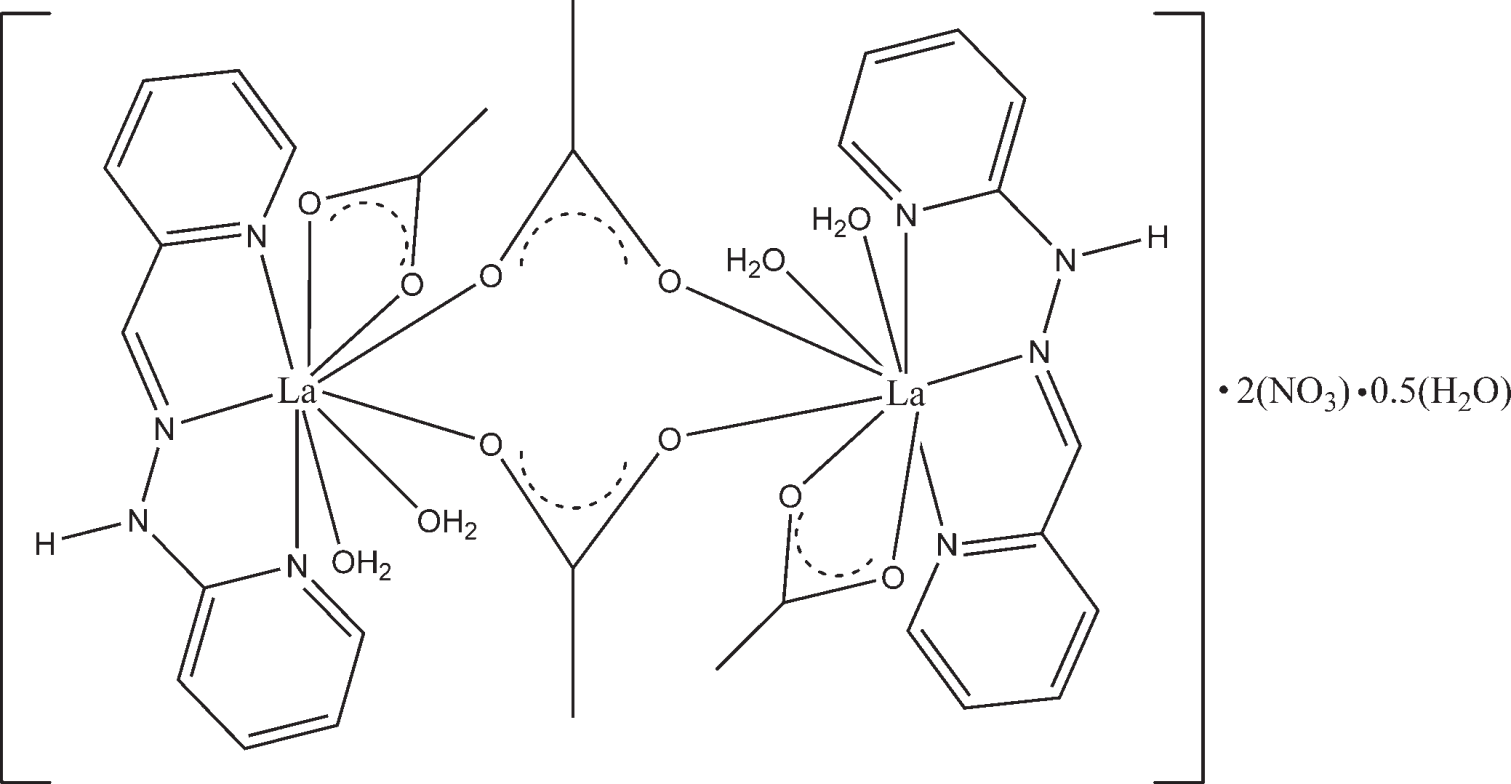


## 2. Structural commentary

**Figure 1:**
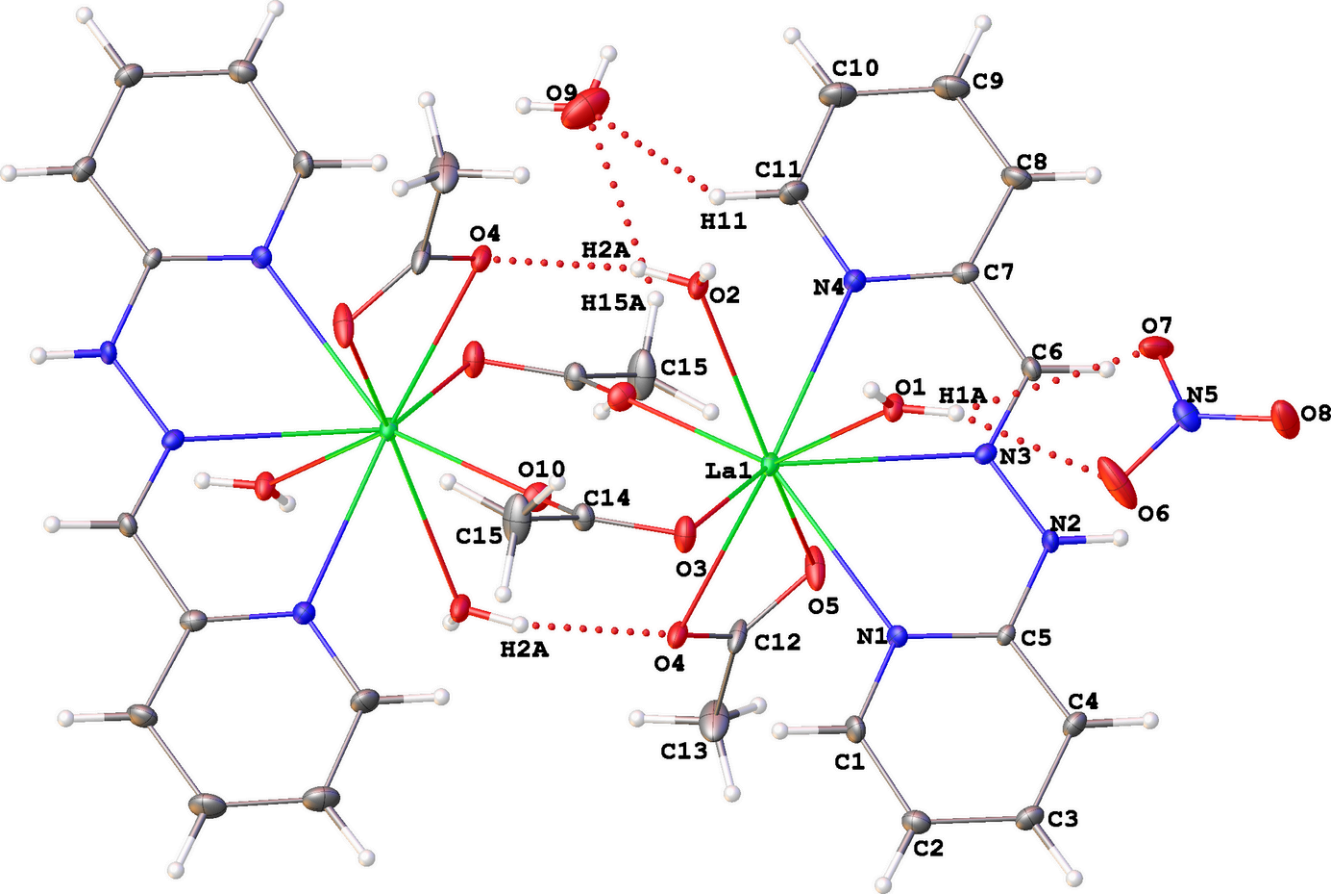
A view of the title compound, showing the atom-numbering scheme for the asymmetric unit. Displacement ellipsoids are drawn at the 30% probability level.

A mixture of the ligand H*L* [1-(pyridin-2-yl)-2-(pyridin-2-yl­methyl­ene)hydrazine], lanthanum nitrate, and acetate salts in a 1:1:3 ratio yields the title compound, which crystallographic studies reveal to be a dicationic binuclear complex with a 1:1:2 stoichiometric ratio. The crystal structure exhibits disorder involving both the ligand and the nitrate group. The site occupancy factors (SOFs) for the two disordered parts of the ligand refine to 0.547 (9) and 0.453 (9). For the nitrate group, the SOFs refine to 0.826 (9) and 0.174 (9). The following analysis of the crystal structure focuses on the major disorder components. The structure of the lanthanum acetato-bridged complex is built from two identical entities {La(H*L*)(*η*^2^-OOCH_3_)(*η*^1^-HO_2_)_2_} bridged by two acetate anions acting in *μ*_2_-OOCH_3_ mode, yielding a binuclear dianionic complex containing two uncoordinated nitrate anions and a partial occupancy non-coordinating water mol­ecule (Figs. 1 ▸ and 2 ▸). Each La^III^ ion is coordinated by one H*L* ligand coordinated through two 2-pyridyl nitro­gen atoms and one azomethine nitro­gen atom. The coordination of the Schiff base forms two five-membered rings (LaNCNN) and (LaNCCN) with bite angles of 59.99 (9) and 59.43 (10)°, respectively, in the major disorder component. Additionally, each La^III^ ion is coordinated by one chelating-bidentate acetate group acting in *η*^2^-OOCH_3_ mode and two chelating-monodentate water mol­ecules acting in *η*^1^-HO_2_ mode. Thus, the La^III^ ions are nine coordinate and their environments are best described as a strongly distorted tricapped trigonal–prismatic geometry. The atoms N4/N3/O5 and O2/O3/O4 define the slanted base faces of the trigonal–tricapped environment. These two planes are twisted and form a dihedral angle of 57.37 (2)°. The three caps are occupied by O1, N1 and N2 atoms. The lanthanum cation is situated 1.320 (4) Å out of the plane defined by the caps O1, N1 and N2 of the polyhedron. The La—N distances (Table 1 ▸) are slightly longer than those found for the analogous complex of the Nd^III^ ion with the same ligand [2.675 (3), 2.637 (2) and 2.639 (2) Å] (Ndiaye-Gueye, Dieng, Thiam, Sow *et al.*, 2017 ▸; Ndiaye-Gueye, Dieng, Thiam, Lo *et al.*, 2017 ▸; Gueye, Dieng *et al.*, 2017 ▸; Gueye, *et al.* 2021 ▸). The La—O distances s fall in the range reported for other carboxyl­ate complexes (Gueye, Moussa *et al.*, 2017 ▸; Bag *et al.*, 2013 ▸; Chen *et al.*, 2014 ▸). The distances for La—OH_2_ are comparable to the values in the complex [{*Ln*(H*L*(*η*^2^-OOCH_3_)_2_(*η*^1^-H_2_O)_2_}{*μ*_2_-OOCH_3_)_2_}{*Ln*(H*L*)(*η*^2^-OOCH_3_)_2_}(*η*^1^-H_2_O)_2_]·2NO_3_, (where *Ln* = Nd or Sm) (Ndiaye-Gueye, Dieng, Thiam, Lo, *et al.*, 2017 ▸). The La^III^⋯La^III^ distance is 4.6696 (6) Å and the value of the bridging angle O3— La1—O10 is 109.21 (5)°. The C6—N3 distance of 1.289 (7) Å is consistent with double-bond character. The bond lengths in the chain C—CH=N—NH—C bridging two pyridine rings are [1.443 (6) Å for PyC—C, 1.289 (7) Å for CH=N, 1.346 (6) Å for N—N and 1.377 (6) Å C—CPy] and are significantly different from the corresponding mean values for this ligand found in the CSD [1.450 (17), 1.283 (15), 1.349 (12) and 1.376 (16) Å, respectively].

## 3. Supra­molecular features

**Figure 3:**
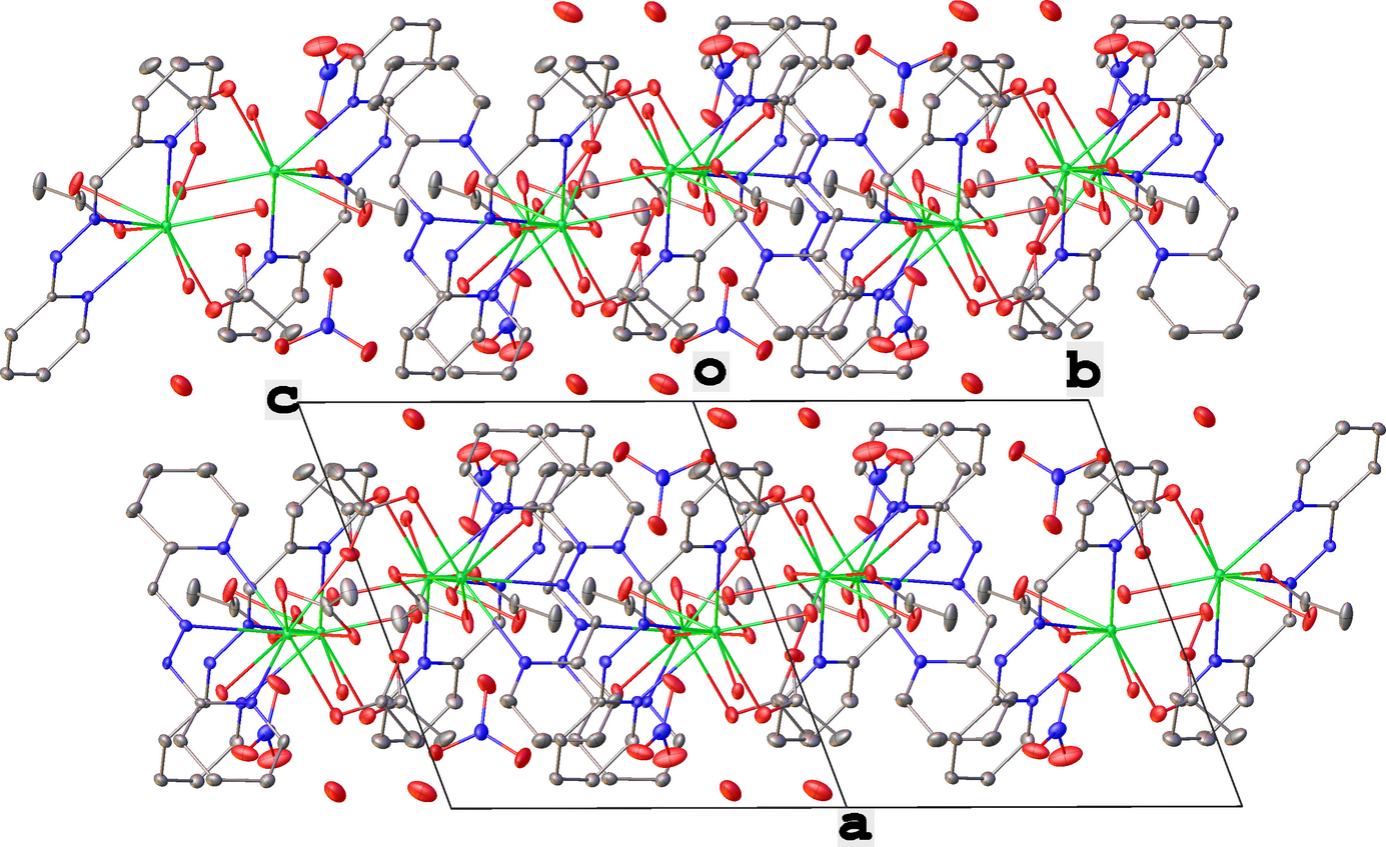
A partial packing plot showing diperiodic sheets that extend parallel to the *bc* plane.

The title complex [{La(H*L*)(*η*^2^-OOCH_3_)(*η*^1^-H_2_O)_2_}{(*μ*_2_-OOCH_3_)_2_}{La(H*L*)(*η*^2^-OOCH_3_)(*η*^1^-H_2_O)_2_}]·2NO_3_·0.5(H_2_O) features both coordinated and solvent water mol­ecules. The unbound solvent water is present at partial occupancy. An intra­molecular hydrogen bond is formed between the OH group of a coordinated water mol­ecule, acting as donor, and an oxygen atom (O7) of a free nitrate group, acting as acceptor (O1—H1*B*⋯O7). In addition, inter­molecular hydrogen bonds involving the OH groups of coordinated water mol­ecules are significant in the construction of the structure. These OH groups act as donors to the nitrate oxygen atoms of free nitrate groups (O1—H1*A*⋯O8^i^ and O2—H2*B*⋯O7^i^; symmetry codes as in Table 1 ▸) and to oxygen atoms of bidentate chelating acetate groups (O2—H2*A*⋯O4^ii^). The NH group of the hydrazine moiety inter­acts with an oxygen atom of a bidentate chelating acetate group, further consolidating the structure through the hydrogen bond N2—H2⋯O5^iii^. Weak inter­molecular C—H⋯O hydrogen bonds are also observed between CH groups and oxygen atoms of the bidentate chelating acetate groups, as summarized in Table 1 ▸. These hydrogen bonds collectively connect the mol­ecules of the complex into a three-dimensional network (Table 2 ▸, Fig. 3 ▸).

## 4. Database survey

A search of the Cambridge Structural Database (CSD version 5.44, updates of September 2023; Groom *et al.*, 2016 ▸) indicated 27 compounds incorporating the ligand 1-(pyridin-2-yl­methyl­idene)-2-(pyridin-2-yl)hydrazine, which has been widely used in coordination chemistry. Seven examples of complexes of the above ligand with *f*-block metal ions are known from the literature: BEHFUS and TESXOH (Gueye, Dieng *et al.*, 2017 ▸), PCPHYB (Baraniak *et al.*, 1976 ▸), TIKDAV and TIKCUO (Ndiaye-Gueye, Dieng, Thiam, Lo, *et al.*, 2017 ▸), ZEFJOM (Gueye, Moussa *et al.*, 2017 ▸), GIJYAD (Ndiaye-Gueye *et al.*, 2022 ▸). Three structures are available for the Ca^2+^ metal ion: NIWLEM, NIWLIQ and NIWLOW (Vantomme, Hafezi *et al.*, 2014 ▸). One Co^2+^ (PAPCOC10; Gerloch, 1966 ▸) and two Mn^2+^ [PEQMAC (Sarr *et al.*, 2018 ▸), SIZPID01 (Diop *et al.*, 2019 ▸)] structures are reported in the CSD. Nine entries for Cu^2+^ are found: DIMLEQ10 and DIMLIU01 (Rojo *et al.*, 1988 ▸), JAWRII (Mesa *et al.*, 1988 ▸), SAHDOU (Mesa *et al.*, 1989 ▸), REJMEY and REJMIC (Ainscough *et al.*, 1996 ▸), QUJTIZ (Chowdhury *et al.*, 2009 ▸) TUSWEK (Mukherjee *et al.*, 2010 ▸), FAFZOF (U-wang *et al.*, 2020 ▸). Five Zn^2+^ structures: GECWAP and GECWIX (Vantomme, Jiang *et al.*, 2014 ▸), SAVQAI and SAVQEM (Dumitru *et al.*, 2005 ▸), SIZPOJ01 (Diop *et al.*, 2019 ▸) are also reported in the CSD.

## 5. Synthesis and crystallization

A mixture of 2-hydrazino­pyridine (1 mmol) and 2-pyridine­carbaldehyde (1 mmol) in ethanol (15 mL) was stirred under reflux for 30 min. A mixture of sodium acetate (3 mmol) and La(NO_3_)_3_·6H_2_O (1 mmol) in ethanol (10 mL) was added to the solution. The mixture was stirred for 30 min and the resulting yellow solution was filtered and the filtrate was kept at 298 K. A yellow powder appeared after one day and was collected by filtration. Recrystallization by slow evaporation of an ethanol solution gave X-ray quality crystals of the compound [C_30_H_40_LaN_8_O_12_]·2NO_3_·0.5H_2_O. Yield 65%. Analysis calculated C, 32.30; H, 3.70; N, 12.56. Found: C, 32.27; H, 3.73; N, 12.52. %.

## 6. Refinement

Crystal data, data collection and structure refinement details are summarized in Table 3 ▸. Hydrogen atoms were found in difference-Fourier maps, but subsequently included in the refinement using riding models, with constrained distances set to 0.93 Å (C*sp*^2^—H), 0.96 Å (*R*CH_3_) and 0.86 Å (N*sp*^2^—H). Water hydrogen atoms were refined using 1,2 and 1,3 distance restraints. *U*_iso_(H) parameters were set to values of either 1.2*U*_eq_ or 1.5*U*_eq_ (*R*CH_3_ and H_2_O only) of the attached atom. To ensure satisfactory refinement for disordered groups in the structure, a combination of constraints and restraints was employed. Constraints (*SHELXL* command EADP) were used to fix *U*^ij^ of overlapping fragments. Restraints were used to ensure the integrity of ill-defined or disordered groups (*SHELXL* commands SAME, DFIX, CHIV, SIMU, and RIGU).

## Supplementary Material

Crystal structure: contains datablock(s) I. DOI: 10.1107/S2056989024012349/pk2715sup1.cif

Structure factors: contains datablock(s) I. DOI: 10.1107/S2056989024012349/pk2715Isup2.hkl

CCDC reference: 2412041

Additional supporting information:  crystallographic information; 3D view; checkCIF report

## Figures and Tables

**Figure 2:**
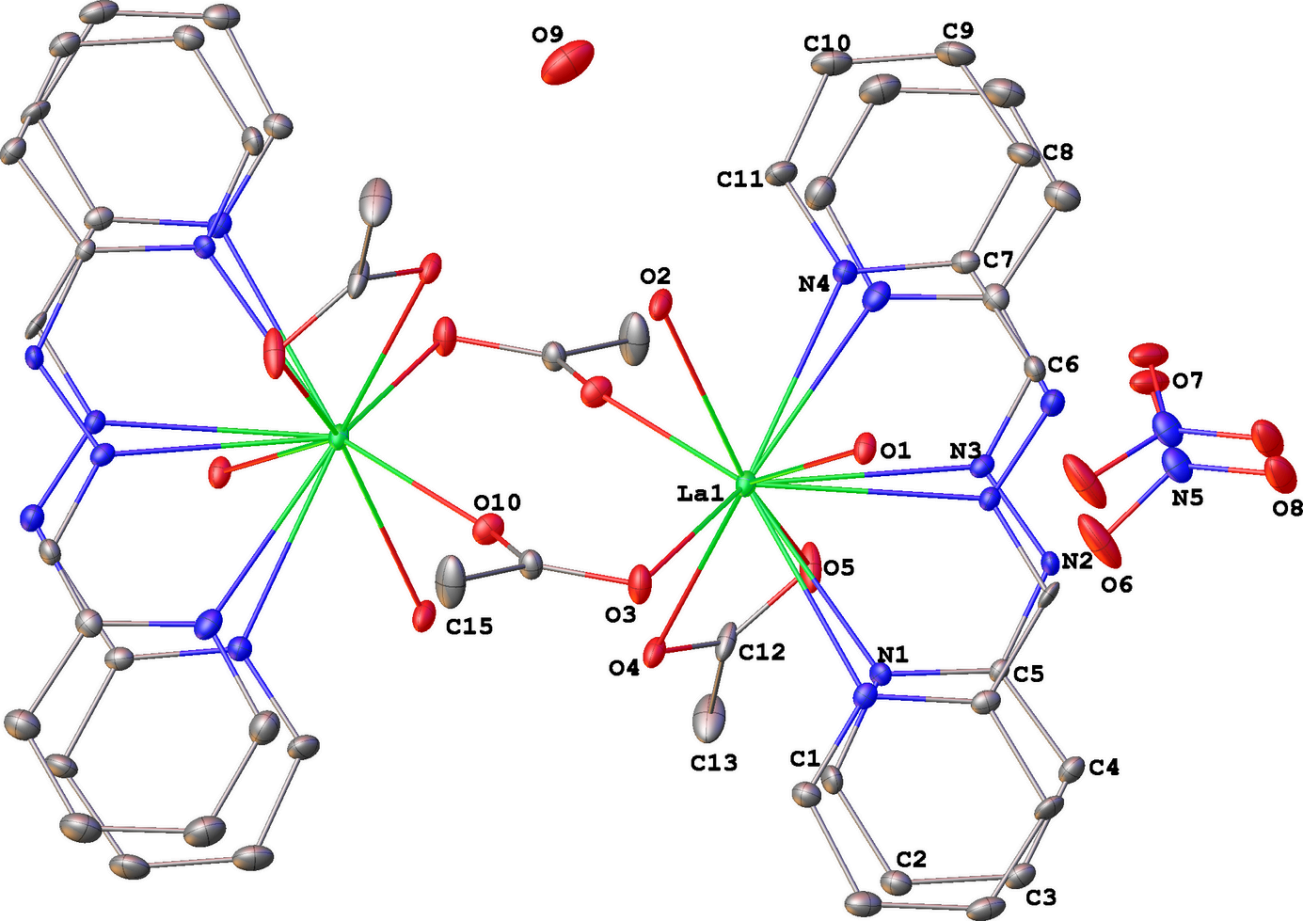
The nature of disorder of the ligand and nitrate anion.

**Table 1:** Selected bond lengths (Å)

La1—O1	2.5659 (14)	La1—O10	2.4814 (14)
La1—O2	2.5395 (15)	La1—N1	2.626 (8)
La1—O3	2.5184 (16)	La1—N3	2.683 (7)
La1—O4	2.5653 (15)	La1—N4	2.768 (6)
La1—O5	2.6073 (16)		

**Table 2:** Hydrogen-bond geometry (Å, °)

*D*—H⋯*A*	*D*—H	H⋯*A*	*D*⋯*A*	*D*—H⋯*A*
O1—H1*A*⋯O6	0.76 (1)	2.27 (2)	2.994 (3)	158 (3)
O1—H1*A*⋯O7	0.76 (1)	2.59 (2)	3.273 (6)	151 (2)
O1—H1*A*⋯O6′	0.76 (1)	2.19 (2)	2.901 (14)	154 (2)
O1—H1*B*⋯O8^i^	0.76 (1)	2.03 (2)	2.793 (4)	176 (3)
O1—H1*B*⋯O8′^i^	0.76 (1)	1.97 (3)	2.73 (2)	171 (3)
O2—H2*A*⋯O4^ii^	0.76 (1)	1.95 (2)	2.6971 (19)	168 (3)
O2—H2*B*⋯O7^i^	0.76 (1)	2.03 (2)	2.786 (4)	171 (3)
O2—H2*B*⋯N5′^i^	0.76 (1)	2.68 (2)	3.419 (16)	164 (3)
O2—H2*B*⋯O7′^i^	0.76 (1)	1.79 (3)	2.54 (2)	167 (3)
C13—H13*A*⋯O7^iii^	0.96	2.53	3.483 (5)	170
C13—H13*A*⋯O7′^iii^	0.96	2.33	3.27 (3)	167
C13—H13*C*⋯O7^iv^	0.96	2.65	3.544 (6)	155
C15—H15*A*⋯O9	0.96	2.62	3.350 (10)	133
C2—H2⋯O6^v^	0.93	2.57	3.420 (6)	153
N2—H2*C*⋯O5^iv^	0.86	2.13	2.898 (7)	149
C11—H11⋯O9	0.93	2.57	3.150 (10)	121
N2′—H2′*A*⋯O5^iv^	0.86	2.30	3.028 (7)	142
C6′—H6′⋯O5^iv^	0.93	2.32	3.067 (9)	138
C10′—H10′⋯O6′^v^	0.93	2.34	3.260 (18)	170
O9—H9*A*⋯O6^vi^	0.76 (2)	2.66 (14)	3.062 (9)	115 (13)
O9—H9*B*⋯O8^vi^	0.76 (2)	2.57 (10)	3.241 (11)	147 (16)

Symmetry codes: (i) 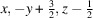; (ii) 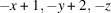; (iii) 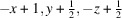; (iv) 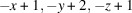; (v) 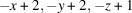; (vi) 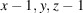.

**Table 3:** Experimental details

Crystal data	
Chemical formula	[La_2_(C_2_H_3_O_2_)_4_(C_11_H_10_N_4_)(H_2_O)_4_](NO_3_)_2_·0.5H_2_O
*M*_r_	1115.55
Crystal system, space group	Monoclinic, *P*2_1_/*c*
Temperature (K)	293
*a*, *b*, *c* (Å)	11.1170 (11), 17.8366 (19), 11.8094 (12)
β (°)	114.213 (3)
*V* (Å^3^)	2135.7 (4)
*Z*	2
Radiation type	Mo *K*α
μ (mm^−1^)	2.06
Crystal size (mm)	0.2 × 0.2 × 0.1

Data collection	
Diffractometer	Bruker X8
Absorption correction	Numerical (*SADABS*; Krause et al., 2015 ▸)
*T*_min_, *T*_max_	0.215, 0.424
No. of measured, independent and observed [*I* > 2σ(*I*)] reflections	72063, 10392, 7368
*R*_int_	0.084
(sin θ/λ)_max_ (Å^−1^)	0.836

Refinement	
*R*[*F*^2^ > 2σ(*F*^2^)], *wR*(*F*^2^), *S*	0.039, 0.064, 1.04
No. of reflections	10392
No. of parameters	451
No. of restraints	781
H-atom treatment	H atoms treated by a mixture of independent and constrained refinement
Δρ_max_, Δρ_min_ (e Å^−3^)	1.26, −0.95

Computer programs: *APEX5* (Bruker, 2023 ▸), *SHELXT* (Sheldrick, 2015*a* ▸), *SHELXL* (Sheldrick, 2015*b* ▸) and *OLEX2* (Dolomanov *et al.*, 2009 ▸).

## supplementary crystallographic information

### Di-µ-acetato-κ4O:O'-bis{(acetato-κ2O,O')tetraaqua[1-(pyridin-2-ylmethylidene-κN)-2-(pyridin-2-yl-κN)hydrazine-κN1]lanthanum(III)} dinitrate hemihydrate Crystal data

**Table:**

[La_2_(C_2_H_3_O_2_)_4_(C_11_H_10_N_4_)(H_2_O)_4_](NO_3_)_2_·0.5H_2_O	*F*(000) = 1106
*M**_r_* = 1115.55	*D*_x_ = 1.735 Mg m^−^^3^
Monoclinic, *P*2_1_/*c*	Mo *K*α radiation, λ = 0.71073 Å
*a* = 11.1170 (11) Å	Cell parameters from 21184 reflections
*b* = 17.8366 (19) Å	θ = 2.7–29.4°
*c* = 11.8094 (12) Å	µ = 2.06 mm^−^^1^
β = 114.213 (3)°	*T* = 293 K
*V* = 2135.7 (4) Å^3^	Block, metallic yellowish yellow
*Z* = 2	0.2 × 0.2 × 0.1 mm

### Di-µ-acetato-κ4O:O'-bis{(acetato-κ2O,O')tetraaqua[1-(pyridin-2-ylmethylidene-κN)-2-(pyridin-2-yl-κN)hydrazine-κN1]lanthanum(III)} dinitrate hemihydrate Data collection

**Table:**

Bruker X8 diffractometer	7368 reflections with *I* > 2σ(*I*)
Detector resolution: 10 pixels mm^-1^	*R*_int_ = 0.084
Single crystals were positioned at 35, 40, 35, and 28 mm from the detector scans	θ_max_ = 36.4°, θ_min_ = 2.2°
Absorption correction: numerical (SADABS; Krause et al., 2015)	*h* = −16→18
*T*_min_ = 0.215, *T*_max_ = 0.424	*k* = −29→29
72063 measured reflections	*l* = −19→19
10392 independent reflections	

### Di-µ-acetato-κ4O:O'-bis{(acetato-κ2O,O')tetraaqua[1-(pyridin-2-ylmethylidene-κN)-2-(pyridin-2-yl-κN)hydrazine-κN1]lanthanum(III)} dinitrate hemihydrate Refinement

**Table:**

Refinement on *F*^2^	Hydrogen site location: mixed
Least-squares matrix: full	H atoms treated by a mixture of independent and constrained refinement
*R*[*F*^2^ > 2σ(*F*^2^)] = 0.039	*w* = 1/[σ^2^(*F*_o_^2^) + (0.0112*P*)^2^ + 1.5461*P*] where *P* = (*F*_o_^2^ + 2*F*_c_^2^)/3
*wR*(*F*^2^) = 0.064	(Δ/σ)_max_ = 0.003
*S* = 1.04	Δρ_max_ = 1.26 e Å^−^^3^
10392 reflections	Δρ_min_ = −0.94 e Å^−^^3^
451 parameters	Extinction correction: SHELXL (Sheldrick, 2015b), Fc^*^=kFc[1+0.001xFc^2^λ^3^/sin(2θ)]^-1/4^
781 restraints	Extinction coefficient: 0.00071 (13)

### Di-µ-acetato-κ4O:O'-bis{(acetato-κ2O,O')tetraaqua[1-(pyridin-2-ylmethylidene-κN)-2-(pyridin-2-yl-κN)hydrazine-κN1]lanthanum(III)} dinitrate hemihydrate Special details

**Table:**

Geometry. All esds (except the esd in the dihedral angle between two l.s. planes) are estimated using the full covariance matrix. The cell esds are taken into account individually in the estimation of esds in distances, angles and torsion angles; correlations between esds in cell parameters are only used when they are defined by crystal symmetry. An approximate (isotropic) treatment of cell esds is used for estimating esds involving l.s. planes.

### Di-µ-acetato-κ4O:O'-bis{(acetato-κ2O,O')tetraaqua[1-(pyridin-2-ylmethylidene-κN)-2-(pyridin-2-yl-κN)hydrazine-κN1]lanthanum(III)} dinitrate hemihydrate Fractional atomic coordinates and isotropic or equivalent isotropic displacement parameters (Å^2^)

**Table:**

	*x*	*y*	*z*	*U*_iso_*/*U*_eq_	Occ. (<1)
La1	0.56737 (2)	0.95905 (2)	0.20467 (2)	0.01520 (3)	
O1	0.71353 (15)	0.84746 (8)	0.31638 (13)	0.0207 (3)	
H1A	0.737 (2)	0.8335 (14)	0.3831 (15)	0.031*	
H1B	0.753 (2)	0.8249 (13)	0.289 (2)	0.031*	
O2	0.52181 (16)	0.84992 (8)	0.05463 (13)	0.0247 (3)	
H2A	0.490 (2)	0.8580 (14)	−0.0147 (14)	0.037*	
H2B	0.574 (2)	0.8205 (13)	0.062 (2)	0.037*	
O3	0.77181 (16)	0.96048 (9)	0.16251 (13)	0.0295 (3)	
O4	0.57600 (14)	1.10181 (8)	0.18261 (12)	0.0218 (3)	
O5	0.46089 (19)	1.06485 (10)	0.28544 (14)	0.0360 (4)	
C12	0.5029 (2)	1.11655 (12)	0.23852 (17)	0.0263 (5)	
C13	0.4664 (3)	1.19661 (14)	0.2475 (2)	0.0449 (7)	
H13A	0.431738	1.218889	0.166320	0.067*	
H13B	0.543322	1.223772	0.300997	0.067*	
H13C	0.400921	1.198418	0.280778	0.067*	
O10	0.37451 (14)	0.99521 (9)	0.01294 (13)	0.0248 (3)	
C14	0.2633 (2)	1.02384 (12)	−0.05024 (18)	0.0207 (4)	
C15	0.1712 (3)	1.04094 (18)	0.0093 (2)	0.0452 (7)	
H15A	0.104232	1.002998	−0.012831	0.068*	
H15B	0.130825	1.088904	−0.018859	0.068*	
H15C	0.219407	1.041944	0.097847	0.068*	
N1	0.7389 (8)	1.0127 (6)	0.4165 (8)	0.0154 (11)	0.547 (8)
C1	0.8351 (10)	1.0594 (7)	0.4194 (8)	0.0221 (14)	0.547 (8)
H1	0.837759	1.072530	0.344276	0.026*	0.547 (8)
C2	0.9305 (7)	1.0891 (5)	0.5271 (6)	0.0234 (12)	0.547 (8)
H2	0.993945	1.122342	0.524696	0.028*	0.547 (8)
C3	0.9276 (6)	1.0672 (4)	0.6394 (5)	0.0228 (10)	0.547 (8)
H3	0.990764	1.085119	0.714130	0.027*	0.547 (8)
C4	0.8317 (6)	1.0194 (3)	0.6390 (5)	0.0207 (10)	0.547 (8)
H4	0.828457	1.004507	0.713141	0.025*	0.547 (8)
C5	0.7388 (6)	0.9932 (4)	0.5261 (5)	0.0123 (9)	0.547 (8)
N2	0.6401 (5)	0.9460 (4)	0.5246 (6)	0.0156 (10)	0.547 (8)
H2C	0.637201	0.932701	0.593393	0.019*	0.547 (8)
N3	0.5482 (6)	0.9206 (4)	0.4163 (6)	0.0154 (10)	0.547 (8)
C6	0.4571 (7)	0.8775 (4)	0.4207 (6)	0.0175 (10)	0.547 (8)
H6	0.455719	0.865341	0.496698	0.021*	0.547 (8)
C7	0.3572 (5)	0.8483 (3)	0.3071 (5)	0.0192 (9)	0.547 (8)
C8	0.2639 (6)	0.7974 (3)	0.3128 (6)	0.0279 (11)	0.547 (8)
H8	0.266593	0.781473	0.388808	0.034*	0.547 (8)
C9	0.1675 (5)	0.7714 (3)	0.2022 (7)	0.0344 (13)	0.547 (8)
H9	0.104301	0.737475	0.202871	0.041*	0.547 (8)
C10	0.1663 (5)	0.7966 (3)	0.0905 (6)	0.0344 (12)	0.547 (8)
H10	0.101955	0.780214	0.015203	0.041*	0.547 (8)
C11	0.2632 (6)	0.8466 (4)	0.0937 (6)	0.0273 (12)	0.547 (8)
H11	0.262204	0.863073	0.018597	0.033*	0.547 (8)
N4	0.3581 (6)	0.8727 (3)	0.1988 (5)	0.0187 (9)	0.547 (8)
N1'	0.3780 (8)	0.8785 (5)	0.2388 (6)	0.0239 (13)	0.453 (8)
C1'	0.2781 (9)	0.8502 (5)	0.1387 (7)	0.0324 (15)	0.453 (8)
H1'	0.271747	0.863508	0.060346	0.039*	0.453 (8)
C2'	0.1839 (8)	0.8019 (5)	0.1470 (8)	0.0418 (16)	0.453 (8)
H2'	0.113781	0.784576	0.076477	0.050*	0.453 (8)
C3'	0.1993 (8)	0.7806 (4)	0.2654 (9)	0.0414 (16)	0.453 (8)
H3'	0.140352	0.746774	0.274681	0.050*	0.453 (8)
C4'	0.2991 (7)	0.8087 (4)	0.3674 (8)	0.0328 (14)	0.453 (8)
H4'	0.308199	0.795176	0.446545	0.039*	0.453 (8)
C5'	0.3880 (7)	0.8582 (4)	0.3514 (7)	0.0202 (11)	0.453 (8)
N2'	0.4887 (7)	0.8877 (4)	0.4527 (6)	0.0202 (12)	0.453 (8)
H2'A	0.493246	0.878841	0.525905	0.024*	0.453 (8)
N3'	0.5807 (7)	0.9306 (5)	0.4385 (7)	0.0145 (12)	0.453 (8)
C6'	0.6765 (8)	0.9552 (6)	0.5382 (8)	0.0174 (13)	0.453 (8)
H6'	0.680870	0.941845	0.615935	0.021*	0.453 (8)
C7'	0.7762 (7)	1.0032 (5)	0.5286 (7)	0.0178 (13)	0.453 (8)
C8'	0.8745 (7)	1.0334 (4)	0.6364 (6)	0.0236 (13)	0.453 (8)
H8'	0.879996	1.020580	0.714666	0.028*	0.453 (8)
C9'	0.9619 (8)	1.0821 (4)	0.6225 (7)	0.0286 (14)	0.453 (8)
H9'	1.027339	1.103938	0.691992	0.034*	0.453 (8)
C10'	0.9537 (9)	1.0992 (6)	0.5056 (8)	0.0290 (16)	0.453 (8)
H10'	1.013469	1.131946	0.495294	0.035*	0.453 (8)
C11'	0.8542 (12)	1.0663 (8)	0.4037 (10)	0.0228 (17)	0.453 (8)
H11'	0.848158	1.078181	0.324895	0.027*	0.453 (8)
N4'	0.7676 (9)	1.0191 (7)	0.4134 (9)	0.0162 (14)	0.453 (8)
N5	0.8120 (3)	0.7717 (2)	0.6227 (3)	0.0278 (7)	0.826 (9)
O6	0.8757 (3)	0.8140 (3)	0.5847 (4)	0.0629 (13)	0.826 (9)
O7	0.6941 (4)	0.7636 (2)	0.5548 (5)	0.0405 (8)	0.826 (9)
O8	0.8623 (4)	0.7415 (2)	0.7271 (3)	0.0445 (10)	0.826 (9)
N5'	0.8000 (16)	0.7501 (9)	0.5993 (15)	0.0278 (7)	0.174 (9)
O6'	0.8688 (15)	0.7717 (12)	0.5480 (17)	0.0629 (13)	0.174 (9)
O7'	0.6816 (18)	0.7484 (13)	0.543 (3)	0.0405 (8)	0.174 (9)
O8'	0.854 (2)	0.7191 (12)	0.7010 (16)	0.0445 (10)	0.174 (9)
O9	0.0421 (9)	0.8898 (6)	−0.1673 (8)	0.062 (3)	0.25
H9A	0.016 (14)	0.907 (8)	−0.232 (6)	0.093*	0.25
H9B	−0.010 (11)	0.861 (7)	−0.170 (13)	0.093*	0.25

### Di-µ-acetato-κ4O:O'-bis{(acetato-κ2O,O')tetraaqua[1-(pyridin-2-ylmethylidene-κN)-2-(pyridin-2-yl-κN)hydrazine-κN1]lanthanum(III)} dinitrate hemihydrate Atomic displacement parameters (Å^2^)

**Table:**

	*U*^11^	*U*^22^	*U*^33^	*U*^12^	*U*^13^	*U*^23^
La1	0.01924 (6)	0.01380 (5)	0.01228 (5)	0.00382 (5)	0.00618 (4)	0.00176 (4)
O1	0.0285 (8)	0.0184 (7)	0.0151 (6)	0.0072 (6)	0.0089 (6)	0.0030 (5)
O2	0.0375 (9)	0.0191 (7)	0.0136 (6)	0.0035 (6)	0.0064 (6)	0.0031 (5)
O3	0.0304 (8)	0.0409 (9)	0.0196 (7)	0.0064 (7)	0.0127 (6)	0.0083 (7)
O4	0.0318 (8)	0.0186 (7)	0.0125 (6)	0.0045 (6)	0.0064 (6)	0.0011 (5)
O5	0.0626 (12)	0.0311 (9)	0.0273 (8)	0.0265 (8)	0.0317 (8)	0.0145 (7)
C12	0.0427 (13)	0.0244 (11)	0.0113 (8)	0.0151 (10)	0.0105 (8)	0.0025 (7)
C13	0.083 (2)	0.0246 (13)	0.0345 (13)	0.0207 (13)	0.0311 (14)	0.0021 (10)
O10	0.0201 (7)	0.0258 (8)	0.0238 (7)	0.0059 (6)	0.0043 (6)	0.0010 (6)
C14	0.0190 (9)	0.0258 (11)	0.0193 (9)	0.0010 (8)	0.0098 (8)	0.0030 (7)
C15	0.0315 (13)	0.078 (2)	0.0347 (13)	0.0181 (14)	0.0220 (11)	0.0165 (14)
N1	0.015 (3)	0.019 (2)	0.0153 (16)	−0.002 (2)	0.0085 (18)	−0.0007 (13)
C1	0.025 (3)	0.027 (3)	0.018 (2)	−0.004 (2)	0.0123 (18)	0.0013 (18)
C2	0.022 (3)	0.023 (3)	0.026 (2)	−0.0031 (18)	0.0110 (18)	−0.0020 (18)
C3	0.018 (3)	0.026 (3)	0.0196 (19)	−0.0020 (19)	0.0038 (17)	−0.0042 (18)
C4	0.019 (2)	0.025 (2)	0.0144 (16)	−0.0004 (18)	0.0029 (17)	−0.0022 (15)
C5	0.011 (2)	0.015 (2)	0.0098 (14)	0.0023 (17)	0.0035 (17)	−0.0005 (13)
N2	0.017 (2)	0.019 (2)	0.012 (2)	−0.0026 (18)	0.0074 (19)	0.0022 (15)
N3	0.016 (3)	0.014 (2)	0.015 (2)	0.0019 (17)	0.0041 (18)	−0.0014 (15)
C6	0.020 (3)	0.018 (2)	0.017 (3)	−0.0020 (19)	0.010 (2)	0.0026 (19)
C7	0.017 (2)	0.0185 (19)	0.022 (2)	−0.0021 (15)	0.0081 (18)	−0.0042 (17)
C8	0.027 (3)	0.026 (2)	0.033 (3)	−0.0114 (19)	0.014 (2)	−0.003 (2)
C9	0.025 (2)	0.035 (3)	0.043 (3)	−0.0134 (19)	0.013 (2)	−0.006 (2)
C10	0.025 (2)	0.034 (2)	0.039 (3)	−0.0091 (18)	0.008 (2)	−0.006 (2)
C11	0.021 (2)	0.030 (2)	0.023 (2)	−0.0069 (17)	0.001 (2)	−0.003 (2)
N4	0.019 (2)	0.0191 (19)	0.017 (2)	−0.0019 (15)	0.0071 (19)	−0.0002 (19)
N1'	0.027 (3)	0.024 (2)	0.019 (3)	0.003 (2)	0.008 (2)	−0.003 (2)
C1'	0.031 (3)	0.039 (3)	0.025 (3)	−0.003 (2)	0.009 (3)	−0.002 (3)
C2'	0.035 (3)	0.046 (4)	0.038 (3)	−0.010 (3)	0.009 (3)	−0.006 (3)
C3'	0.035 (4)	0.041 (4)	0.046 (4)	−0.013 (3)	0.015 (3)	−0.003 (3)
C4'	0.030 (3)	0.032 (3)	0.038 (3)	−0.006 (2)	0.015 (3)	−0.001 (3)
C5'	0.021 (3)	0.020 (2)	0.022 (3)	0.0015 (19)	0.011 (2)	0.000 (2)
N2'	0.020 (3)	0.025 (3)	0.016 (3)	−0.002 (2)	0.007 (2)	0.000 (2)
N3'	0.015 (3)	0.016 (3)	0.012 (3)	0.001 (2)	0.005 (2)	0.0004 (19)
C6'	0.018 (3)	0.023 (3)	0.0060 (19)	0.002 (2)	−0.001 (2)	−0.0032 (19)
C7'	0.013 (3)	0.017 (3)	0.020 (2)	0.002 (2)	0.004 (2)	−0.0022 (17)
C8'	0.018 (3)	0.030 (3)	0.0148 (19)	−0.005 (2)	−0.002 (2)	−0.005 (2)
C9'	0.022 (3)	0.025 (3)	0.030 (3)	−0.004 (2)	0.002 (2)	−0.006 (2)
C10'	0.026 (3)	0.023 (3)	0.033 (3)	−0.002 (2)	0.007 (2)	−0.002 (2)
C11'	0.021 (3)	0.021 (3)	0.026 (3)	−0.003 (2)	0.010 (2)	−0.001 (2)
N4'	0.014 (3)	0.018 (3)	0.015 (2)	0.004 (2)	0.005 (2)	−0.0002 (17)
N5	0.0304 (12)	0.0219 (16)	0.0343 (15)	0.0066 (12)	0.0165 (12)	0.0098 (12)
O6	0.0440 (13)	0.064 (3)	0.090 (2)	0.0082 (16)	0.0369 (15)	0.050 (2)
O7	0.0407 (14)	0.025 (2)	0.0347 (14)	−0.0131 (11)	−0.0054 (11)	0.0036 (14)
O8	0.0282 (11)	0.059 (3)	0.0363 (16)	−0.0100 (16)	0.0031 (13)	0.0233 (15)
N5'	0.0304 (12)	0.0219 (16)	0.0343 (15)	0.0066 (12)	0.0165 (12)	0.0098 (12)
O6'	0.0440 (13)	0.064 (3)	0.090 (2)	0.0082 (16)	0.0369 (15)	0.050 (2)
O7'	0.0407 (14)	0.025 (2)	0.0347 (14)	−0.0131 (11)	−0.0054 (11)	0.0036 (14)
O8'	0.0282 (11)	0.059 (3)	0.0363 (16)	−0.0100 (16)	0.0031 (13)	0.0233 (15)
O9	0.047 (5)	0.069 (7)	0.047 (5)	0.007 (5)	−0.004 (4)	−0.017 (4)

### Di-µ-acetato-κ4O:O'-bis{(acetato-κ2O,O')tetraaqua[1-(pyridin-2-ylmethylidene-κN)-2-(pyridin-2-yl-κN)hydrazine-κN1]lanthanum(III)} dinitrate hemihydrate Geometric parameters (Å, º)

**Table:**

La1—O1	2.5659 (14)	C7—C8	1.400 (7)
La1—O2	2.5395 (15)	C7—N4	1.355 (6)
La1—O3	2.5184 (16)	C8—H8	0.9300
La1—O4	2.5653 (15)	C8—C9	1.386 (6)
La1—O5	2.6073 (16)	C9—H9	0.9300
La1—O10	2.4814 (14)	C9—C10	1.388 (7)
La1—N1	2.626 (8)	C10—H10	0.9300
La1—N3	2.683 (7)	C10—C11	1.388 (7)
La1—N4	2.768 (6)	C11—H11	0.9300
La1—N1'	2.712 (8)	C11—N4	1.339 (6)
La1—N3'	2.752 (8)	N1'—C1'	1.345 (8)
La1—N4'	2.771 (9)	N1'—C5'	1.338 (7)
O1—H1A	0.763 (14)	C1'—H1'	0.9300
O1—H1B	0.760 (14)	C1'—C2'	1.389 (10)
O2—H2A	0.761 (14)	C2'—H2'	0.9300
O2—H2B	0.761 (14)	C2'—C3'	1.390 (9)
O3—C14^i^	1.251 (2)	C3'—H3'	0.9300
O4—C12	1.267 (3)	C3'—C4'	1.356 (8)
O5—C12	1.260 (3)	C4'—H4'	0.9300
C12—C13	1.500 (3)	C4'—C5'	1.394 (8)
C13—H13A	0.9600	C5'—N2'	1.365 (6)
C13—H13B	0.9600	N2'—H2'A	0.8600
C13—H13C	0.9600	N2'—N3'	1.340 (8)
O10—C14	1.261 (2)	N3'—C6'	1.298 (7)
C14—C15	1.491 (3)	C6'—H6'	0.9300
C15—H15A	0.9600	C6'—C7'	1.441 (8)
C15—H15B	0.9600	C7'—C8'	1.401 (8)
C15—H15C	0.9600	C7'—N4'	1.355 (10)
N1—C1	1.345 (7)	C8'—H8'	0.9300
N1—C5	1.340 (8)	C8'—C9'	1.363 (8)
C1—H1	0.9300	C9'—H9'	0.9300
C1—C2	1.385 (9)	C9'—C10'	1.379 (9)
C2—H2	0.9300	C10'—H10'	0.9300
C2—C3	1.396 (7)	C10'—C11'	1.386 (11)
C3—H3	0.9300	C11'—H11'	0.9300
C3—C4	1.364 (6)	C11'—N4'	1.319 (9)
C4—H4	0.9300	N5—O6	1.238 (3)
C4—C5	1.390 (6)	N5—O7	1.233 (4)
C5—N2	1.377 (6)	N5—O8	1.248 (3)
N2—H2C	0.8600	N5'—O6'	1.216 (14)
N2—N3	1.346 (6)	N5'—O7'	1.208 (15)
N3—C6	1.289 (7)	N5'—O8'	1.231 (15)
C6—H6	0.9300	O9—H9A	0.761 (16)
C6—C7	1.443 (6)	O9—H9B	0.762 (16)
			
O1—La1—O5	131.31 (5)	N1—C1—C2	124.2 (6)
O1—La1—N1	74.6 (2)	C2—C1—H1	117.9
O1—La1—N3	66.31 (17)	C1—C2—H2	121.4
O1—La1—N4	85.96 (14)	C1—C2—C3	117.2 (5)
O1—La1—N1'	83.07 (18)	C3—C2—H2	121.4
O1—La1—N3'	65.1 (2)	C2—C3—H3	120.2
O1—La1—N4'	74.6 (3)	C4—C3—C2	119.6 (5)
O2—La1—O1	70.56 (5)	C4—C3—H3	120.2
O2—La1—O4	134.03 (4)	C3—C4—H4	120.5
O2—La1—O5	145.03 (6)	C3—C4—C5	119.1 (5)
O2—La1—N1	143.4 (2)	C5—C4—H4	120.5
O2—La1—N3	112.63 (15)	N1—C5—C4	123.0 (5)
O2—La1—N4	68.32 (13)	N1—C5—N2	117.5 (5)
O2—La1—N1'	75.71 (16)	N2—C5—C4	119.5 (5)
O2—La1—N3'	118.17 (18)	C5—N2—H2C	119.7
O2—La1—N4'	140.7 (3)	N3—N2—C5	120.5 (5)
O3—La1—O1	71.15 (5)	N3—N2—H2C	119.7
O3—La1—O2	78.91 (6)	N2—N3—La1	118.6 (4)
O3—La1—O4	84.00 (5)	C6—N3—La1	123.6 (4)
O3—La1—O5	130.31 (6)	C6—N3—N2	117.8 (6)
O3—La1—N1	79.82 (16)	N3—C6—H6	120.1
O3—La1—N3	127.56 (15)	N3—C6—C7	119.8 (5)
O3—La1—N4	144.90 (14)	C7—C6—H6	120.1
O3—La1—N1'	148.59 (18)	C8—C7—C6	119.5 (5)
O3—La1—N3'	121.26 (17)	N4—C7—C6	117.4 (4)
O3—La1—N4'	73.2 (2)	N4—C7—C8	123.1 (4)
O4—La1—O1	141.26 (5)	C7—C8—H8	120.9
O4—La1—O5	50.23 (5)	C9—C8—C7	118.2 (5)
O4—La1—N1	72.1 (2)	C9—C8—H8	120.9
O4—La1—N3	111.64 (16)	C8—C9—H9	120.3
O4—La1—N4	128.03 (14)	C8—C9—C10	119.4 (5)
O4—La1—N1'	127.18 (18)	C10—C9—H9	120.3
O4—La1—N3'	107.23 (19)	C9—C10—H10	120.8
O4—La1—N4'	69.9 (2)	C9—C10—C11	118.5 (5)
O5—La1—N1	69.4 (2)	C11—C10—H10	120.8
O5—La1—N3	67.84 (17)	C10—C11—H11	118.1
O5—La1—N4	84.78 (14)	N4—C11—C10	123.8 (5)
O5—La1—N1'	80.33 (18)	N4—C11—H11	118.1
O5—La1—N3'	67.2 (2)	C7—N4—La1	119.1 (3)
O5—La1—N4'	73.4 (2)	C11—N4—La1	123.4 (4)
O10—La1—O1	143.00 (5)	C11—N4—C7	117.1 (5)
O10—La1—O2	73.27 (5)	C1'—N1'—La1	118.7 (5)
O10—La1—O3	109.21 (5)	C5'—N1'—La1	122.6 (4)
O10—La1—O4	72.76 (5)	C5'—N1'—C1'	118.2 (7)
O10—La1—O5	78.09 (5)	N1'—C1'—H1'	118.5
O10—La1—N1	142.4 (2)	N1'—C1'—C2'	123.0 (7)
O10—La1—N3	123.20 (15)	C2'—C1'—H1'	118.5
O10—La1—N4	73.67 (12)	C1'—C2'—H2'	121.5
O10—La1—N1'	80.82 (16)	C1'—C2'—C3'	117.1 (6)
O10—La1—N3'	129.37 (18)	C3'—C2'—H2'	121.5
O10—La1—N4'	142.2 (3)	C2'—C3'—H3'	119.6
N1—La1—N3	60.63 (17)	C4'—C3'—C2'	120.7 (6)
N1—La1—N4	120.08 (16)	C4'—C3'—H3'	119.6
N3—La1—N4	59.61 (13)	C3'—C4'—H4'	120.6
N1'—La1—N3'	58.56 (16)	C3'—C4'—C5'	118.8 (6)
N1'—La1—N4'	117.50 (19)	C5'—C4'—H4'	120.6
N3'—La1—N4'	59.0 (2)	N1'—C5'—C4'	122.1 (6)
La1—O1—H1A	131.3 (18)	N1'—C5'—N2'	118.0 (6)
La1—O1—H1B	123.4 (18)	N2'—C5'—C4'	119.9 (6)
H1A—O1—H1B	105 (2)	C5'—N2'—H2'A	119.8
La1—O2—H2A	118.5 (19)	N3'—N2'—C5'	120.3 (6)
La1—O2—H2B	122 (2)	N3'—N2'—H2'A	119.8
H2A—O2—H2B	105 (2)	N2'—N3'—La1	120.0 (4)
C14^i^—O3—La1	107.31 (13)	C6'—N3'—La1	122.3 (6)
C12—O4—La1	95.42 (13)	C6'—N3'—N2'	117.6 (7)
C12—O5—La1	93.60 (13)	N3'—C6'—H6'	120.0
O4—C12—C13	118.9 (2)	N3'—C6'—C7'	120.1 (7)
O5—C12—O4	120.66 (19)	C7'—C6'—H6'	120.0
O5—C12—C13	120.4 (2)	C8'—C7'—C6'	119.7 (6)
C12—C13—H13A	109.5	N4'—C7'—C6'	117.6 (6)
C12—C13—H13B	109.5	N4'—C7'—C8'	122.6 (6)
C12—C13—H13C	109.5	C7'—C8'—H8'	121.1
H13A—C13—H13B	109.5	C9'—C8'—C7'	117.7 (6)
H13A—C13—H13C	109.5	C9'—C8'—H8'	121.1
H13B—C13—H13C	109.5	C8'—C9'—H9'	119.9
La1—O10—La1^i^	116.24 (6)	C8'—C9'—C10'	120.2 (6)
C14—O10—La1^i^	83.51 (11)	C10'—C9'—H9'	119.9
C14—O10—La1	156.33 (14)	C9'—C10'—H10'	120.8
O3^i^—C14—La1^i^	50.25 (10)	C9'—C10'—C11'	118.5 (7)
O3^i^—C14—O10	121.44 (19)	C11'—C10'—H10'	120.8
O3^i^—C14—C15	118.55 (19)	C10'—C11'—H11'	118.5
O10—C14—La1^i^	72.88 (11)	N4'—C11'—C10'	123.1 (8)
O10—C14—C15	120.00 (19)	N4'—C11'—H11'	118.5
C15—C14—La1^i^	161.51 (17)	C7'—N4'—La1	120.9 (5)
C14—C15—H15A	109.5	C11'—N4'—La1	121.2 (6)
C14—C15—H15B	109.5	C11'—N4'—C7'	117.9 (8)
C14—C15—H15C	109.5	O6—N5—O8	122.0 (3)
H15A—C15—H15B	109.5	O7—N5—O6	116.7 (3)
H15A—C15—H15C	109.5	O7—N5—O8	121.2 (4)
H15B—C15—H15C	109.5	O6'—N5'—O8'	118.5 (17)
C1—N1—La1	120.6 (5)	O7'—N5'—O6'	120.1 (19)
C5—N1—La1	122.5 (4)	O7'—N5'—O8'	120.4 (19)
C5—N1—C1	116.8 (6)	H9A—O9—H9B	104 (3)
N1—C1—H1	117.9		
			
La1—O4—C12—O5	3.1 (2)	C7—C8—C9—C10	−0.2 (8)
La1—O4—C12—C13	−176.59 (19)	C8—C7—N4—La1	−172.4 (4)
La1—O5—C12—O4	−3.0 (2)	C8—C7—N4—C11	0.8 (9)
La1—O5—C12—C13	176.64 (19)	C8—C9—C10—C11	0.6 (9)
La1—O10—C14—La1^i^	148.0 (3)	C9—C10—C11—N4	−0.4 (10)
La1^i^—O10—C14—O3^i^	13.4 (2)	C10—C11—N4—La1	172.5 (5)
La1—O10—C14—O3^i^	161.5 (2)	C10—C11—N4—C7	−0.3 (10)
La1^i^—O10—C14—C15	−165.5 (2)	N4—C7—C8—C9	−0.6 (8)
La1—O10—C14—C15	−17.4 (5)	N1'—C1'—C2'—C3'	−2.6 (13)
La1—N1—C1—C2	−178.9 (9)	N1'—C5'—N2'—N3'	−5.7 (11)
La1—N1—C5—C4	177.9 (5)	C1'—N1'—C5'—C4'	0.3 (11)
La1—N1—C5—N2	−3.2 (10)	C1'—N1'—C5'—N2'	−179.3 (7)
La1—N3—C6—C7	−1.0 (9)	C1'—C2'—C3'—C4'	2.6 (12)
La1—N1'—C1'—C2'	173.1 (7)	C2'—C3'—C4'—C5'	−1.3 (11)
La1—N1'—C5'—C4'	−171.4 (5)	C3'—C4'—C5'—N1'	−0.2 (11)
La1—N1'—C5'—N2'	9.0 (9)	C3'—C4'—C5'—N2'	179.4 (7)
La1—N3'—C6'—C7'	4.1 (12)	C4'—C5'—N2'—N3'	174.7 (8)
N1—C1—C2—C3	1.8 (15)	C5'—N1'—C1'—C2'	1.2 (13)
N1—C5—N2—N3	0.3 (10)	C5'—N2'—N3'—La1	−0.2 (10)
C1—N1—C5—C4	0.5 (12)	C5'—N2'—N3'—C6'	−177.8 (8)
C1—N1—C5—N2	179.4 (8)	N2'—N3'—C6'—C7'	−178.3 (8)
C1—C2—C3—C4	−1.1 (11)	N3'—C6'—C7'—C8'	175.9 (9)
C2—C3—C4—C5	0.3 (9)	N3'—C6'—C7'—N4'	−2.0 (13)
C3—C4—C5—N1	0.1 (10)	C6'—C7'—C8'—C9'	−176.0 (7)
C3—C4—C5—N2	−178.8 (6)	C6'—C7'—N4'—La1	−1.0 (12)
C4—C5—N2—N3	179.3 (6)	C6'—C7'—N4'—C11'	176.2 (10)
C5—N1—C1—C2	−1.5 (15)	C7'—C8'—C9'—C10'	−1.3 (11)
C5—N2—N3—La1	2.5 (8)	C8'—C7'—N4'—La1	−178.9 (6)
C5—N2—N3—C6	−179.0 (7)	C8'—C7'—N4'—C11'	−1.7 (15)
N2—N3—C6—C7	−179.4 (6)	C8'—C9'—C10'—C11'	0.7 (14)
N3—C6—C7—C8	175.6 (6)	C9'—C10'—C11'—N4'	−0.6 (18)
N3—C6—C7—N4	−5.1 (9)	C10'—C11'—N4'—La1	178.2 (10)
C6—C7—C8—C9	178.7 (5)	C10'—C11'—N4'—C7'	1.0 (18)
C6—C7—N4—La1	8.3 (7)	N4'—C7'—C8'—C9'	1.8 (12)
C6—C7—N4—C11	−178.5 (6)		

Symmetry code: (i) −*x*+1, −*y*+2, −*z*.

### Di-µ-acetato-κ4O:O'-bis{(acetato-κ2O,O')tetraaqua[1-(pyridin-2-ylmethylidene-κN)-2-(pyridin-2-yl-κN)hydrazine-κN1]lanthanum(III)} dinitrate hemihydrate Hydrogen-bond geometry (Å, º)

**Table:**

*D*—H···*A*	*D*—H	H···*A*	*D*···*A*	*D*—H···*A*
O1—H1*A*···O6	0.76 (1)	2.27 (2)	2.994 (3)	158 (3)
O1—H1*A*···O7	0.76 (1)	2.59 (2)	3.273 (6)	151 (2)
O1—H1*A*···O6′	0.76 (1)	2.19 (2)	2.901 (14)	154 (2)
O1—H1*B*···O8^ii^	0.76 (1)	2.03 (2)	2.793 (4)	176 (3)
O1—H1*B*···O8′^ii^	0.76 (1)	1.97 (3)	2.73 (2)	171 (3)
O2—H2*A*···O4^i^	0.76 (1)	1.95 (2)	2.6971 (19)	168 (3)
O2—H2*B*···O7^ii^	0.76 (1)	2.03 (2)	2.786 (4)	171 (3)
O2—H2*B*···N5′^ii^	0.76 (1)	2.68 (2)	3.419 (16)	164 (3)
O2—H2*B*···O7′^ii^	0.76 (1)	1.79 (3)	2.54 (2)	167 (3)
C13—H13*A*···O7^iii^	0.96	2.53	3.483 (5)	170
C13—H13*A*···O7′^iii^	0.96	2.33	3.27 (3)	167
C13—H13*C*···O7^iv^	0.96	2.65	3.544 (6)	155
C15—H15*A*···O9	0.96	2.62	3.350 (10)	133
C2—H2···O6^v^	0.93	2.57	3.420 (6)	153
N2—H2*C*···O5^iv^	0.86	2.13	2.898 (7)	149
C11—H11···O9	0.93	2.57	3.150 (10)	121
N2′—H2′*A*···O5^iv^	0.86	2.30	3.028 (7)	142
C6′—H6′···O5^iv^	0.93	2.32	3.067 (9)	138
C10′—H10′···O6′^v^	0.93	2.34	3.260 (18)	170
O9—H9*A*···O6^vi^	0.76 (2)	2.66 (14)	3.062 (9)	115 (13)
O9—H9*B*···O8^vi^	0.76 (2)	2.57 (10)	3.241 (11)	147 (16)

Symmetry codes: (i) −*x*+1, −*y*+2, −*z*; (ii) *x*, −*y*+3/2, *z*−1/2; (iii) −*x*+1, *y*+1/2, −*z*+1/2; (iv) −*x*+1, −*y*+2, −*z*+1; (v) −*x*+2, −*y*+2, −*z*+1; (vi) *x*−1, *y*, *z*−1.
